# Flotillins in membrane trafficking and physiopathology

**DOI:** 10.1111/boc.202400134

**Published:** 2025-01-29

**Authors:** Stéphane Bodin, Hadeer Elhabashy, Ewan Macdonald, Dominic Winter, Cécile Gauthier‐Rouvière

**Affiliations:** ^1^ CRBM (Centre de Recherche en Biologie cellulaire de Montpellier), BIOLuM University of Montpellier, CNRS UMR 5237 Montpellier France; ^2^ Department of Protein Evolution Max Planck Institute for Biology Tübingen Germany; ^3^ Department of Computer Science University of Tübingen Tübingen Germany; ^4^ Institute for Bioinformatics and Medical Informatics University of Tübingen Tübingen Germany; ^5^ Institute of Biochemistry and Molecular Biology University of Bonn Bonn Germany

**Keywords:** flotillins, membrane scaffolding, sphingolipids, sphingosine, vesicular trafficking

## Abstract

Flotillin 1 and 2 are highly conserved and homologous members of the stomatin, prohibitin, flotillin, HflK/C (SPFH) family. These ubiquitous proteins assemble into hetero‐oligomers at the cytoplasmic membrane in sphingolipid‐enriched domains. Flotillins play crucial roles in various cellular processes, likely by concentrating sphingosine. They primarily act as scaffolding protein complexes within membrane microdomains (also called lipid rafts) and induce endocytosis and trafficking. Their diverse cargos in the upregulated flotillin–induced trafficking (UFIT) pathway, including tyrosine kinase receptors, adhesion molecules, and neurotransmitter receptors, link them to a wide range of cellular processes and diseases. Consequently, flotillin upregulation has been associated with various pathological conditions such as cancer, metabolic disorders, and neurodegenerative diseases. Flotillins may also be co‐opted by pathogens to facilitate their entry and growth within host cells.

In this review, we examined recent advancements in elucidating the structure and functions of the flotillin protein complex, including its implications in favoring the generation of sphingosine 1‐phosphate, an essential bioactive lipid. We emphasized how the recent cryo‐electron microscopy (cryo‐EM) structure of a truncated cone‐shaped cage composed of 22 copies of flotillin 1 and 2 subunits has enhanced our understanding of the flotillin complex organization within membrane microdomains and its role in membrane remodeling. We also explored how flotillin upregulation can perturb endosomal trafficking and contribute to various pathologies.

A comprehensive understanding of flotillin oligomer organization and function is crucial to developing targeted therapies for diseases associated with flotillin overexpression.

## Graphical abstract

Flotillin 1 and 2 scaffold sphingolipid‐rich membrane microdomains involved in endocytosis and  endolysosome maturation through the generation of sphingosine 1‐phosphate (S1P) by sphingosine kinase 2 (SphK2). Hence they deregulate protein cargo trafficking and influence cell signaling and cellular invasion. Flotillins overexpression is linked to cancer and neurodegenerative diseases. Recent advances in flotillin oligomer structure reveal insights into their role in membrane dynamics.

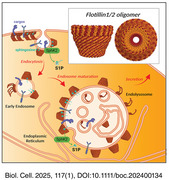

**Abbreviations**
APP: amyloid precursor protein
βA: beta amyloid
CRAC: cholesterol‐recognizing amino acid consensus
CC: coiled‐coil domain
CLEM: Correlative light electron microscopy
CTD: C‐terminal domain
cryo‐EM: cryo‐electron microscopy
DAT: dopamine transporter
LDL: low‐density lipoprotein
MPP1: membrane palmitoylated protein 1
MVEs: multivesicular endosomes
NPC1: Niemann‐Pick C1
NPC1L1: Niemann‐Pick C1‐like 1
OMDP: outer membrane dome protein complex
PKC: Protein Kinase C
SoHo: sorbin homology
S1P: sphingosine 1‐phosphate
SphK2: sphingosine kinase
SPFH: stomatin, prohibitin, flotillin, HflK/C
UFIT: upregulated flotillin‐induced trafficking

## INTRODUCTION

Cell membrane remodeling is driven by a diverse array of proteins that participate in bending, tubulation, fission, scission, and cargo loading. In the past thirty years, particular attention has been given to proteins that scaffold membrane microdomains, locally concentrating bioactive lipids, and protein complexes that can deform membranes and/or contribute to the sorting of protein cargos. Flotillins belong to this category. They were first identified as lipid raft‐enriched proteins in 1997 (Bickel et al., 1997). Their roles in cellular mechanisms (e.g., signaling) and their involvement in cancer, neurodegenerative, and infectious diseases are well established (for review see (Angelopoulou et al., 2020; Gauthier‐Rouvière et al., 2020). However, the exact mechanisms by which flotillins organize proteins and lipids to remodel cell membranes and influence vesicular trafficking remain elusive. This uncertainty is partly due to the controversy that surrounded their role in endocytosis and that caused flotillins to be overlooked by many groups in the membrane trafficking field. However, in the past decade, studies have clarified their function, leading to renewed interest in flotillin roles across various pathophysiological processes. Recent advances in cellular imaging and mass spectrometry have opened a new era in our understanding of metazoan flotillin organization and function at the membrane (Fu & MacKinnon, 2024; Lu et al., 2024; Singh et al., 2022).

The aim of this review is to summarize the current knowledge on mammalian flotillins, with a particular focus on their involvement in membrane trafficking and their implication in cell physiology and pathology.

## INVOLVEMENT OF FLOTILLINS IN PHYSIOLOGICAL CELL MECHANISMS

Flotillins were simultaneously discovered as proteins enriched in lipid rafts isolated as detergent‐resistant membranes from the plasma membrane of lung cells (Bickel et al., 1997) and as proteins (called reggies) upregulated in retinal ganglion cells after nerve injury (Schulte et al., 1997).

Flotillins belong to the stomatin‐prohibitin‐flotillin‐HflC/K (SPFH) protein superfamily due to the SPFH domain they share with all members of this family. They exist as two very similar proteins (flotillin 1 and flotillin 2) that are highly conserved in metazoans, although absent in *Caenorhabditis elegans*. They are encoded by two genes that are highly conserved among species (e.g., 98% of identity between the mouse and human flotillin 1 amino acid sequences). In mammals, flotillin 1 and 2 exhibit approximately 48% of sequence identity. They are expressed in all tissues but are enriched particularly in the nervous system, muscle/adipose tissue, and erythrocytes (Bickel et al., 1997; Volonté et al., 1999). Flotillin‐like proteins have been identified in plants, bacteria, and fungi (Rivera‐Milla et al., 2006), but not in budding yeast and *C. elegans*.

Flotillin 1 and 2 form hetero‐oligomers, and the absence of one flotillin often results in the destabilization and subsequent degradation of the other (Babuke et al., 2009; Frick et al., 2007; Guillaume et al., 2013; Langhorst et al., 2008). This interdependence underscores the essential role of both proteins in maintaining their functional integrity.

Flotillin cellular distribution and functions are intricately linked to their expression levels that can vary significantly between normal and pathological cells. In healthy cells, flotillin 1 and flotillin 2 expression levels are mostly moderate to those observed in pathological cells. For instance, although their lower expression was reported in invasive neuroblastoma (Tomiyama et al., 2014), flotillins overexpression is observed and considered as a marker of poor prognosis in most solid tumors. When moderately expressed, flotillins are predominantly localized at the plasma membrane, particularly within microdomains, also known as lipid rafts, that are specialized microdomains enriched in cholesterol and sphingolipids. These domains serve as platforms for the organization of various molecular complexes. Flotillins are also found in endosomes, Golgi apparatus, endoplasmic reticulum, and extracellular vesicles (Dermine et al., 2001; Langhorst et al., 2005; Planchon et al., 2018; Stuermer, 2010). The localization of flotillins in these microdomains is critical for their establishment and their functional roles in adhesion, cell signaling, lipid homeostasis, protein sorting, and membrane trafficking (Bodin et al., 2014; Gauthier‐Rouvière et al., 2020; Taulet et al., 2009).

As scaffolding proteins, flotillins facilitate the assembly of signaling complexes by bringing together various signaling molecules, thereby enhancing the efficiency and specificity of signal transmission. This role is particularly evident in the context of receptor tyrosine kinases and G‐protein‐coupled receptors, where flotillins modulate downstream signaling pathways involved in cell growth, differentiation, and survival (Sugawara et al., 2007; Tomasovic et al., 2012). Flotillin domains at the plasma membrane also facilitate the assembly and stabilization of adhesion molecules, such as cadherins and integrins, thus regulating cell‐cell adhesion and migration (Banning et al., 2018; Guillaume et al., 2013). Flotillins are also implicated in clathrin‐independent endocytosis (Frick et al., 2007; Glebov et al., 2006), a pathway that is crucial for the internalization of some receptors, lipids, and pathogens. By mediating this endocytic route, flotillins contribute to the regulation of membrane composition and receptor recycling, which are vital for cellular homeostasis and responsiveness to extracellular signals (Genest et al., 2022). Flotillins bind to actin directly and this stabilizes the flotillin domains in the membrane (Langhorst et al., 2007). Furthermore, flotillins play a key role in the regulation of the actin cytoskeleton. They influence cytoskeletal dynamics by interacting with actin‐binding proteins, thereby affecting cell shape, motility, and adhesion (Affentranger et al., 2011; Langhorst et al., 2007; Munderloh et al., 2009; Neumann‐Giesen et al., 2007; Rossy et al., 2009). This regulatory function is critical, for instance, during cell migration, wound healing, and immune responses.

## ABERRANT FLOTILLIN EXPRESSION AND DISTRIBUTION ARE LINKED TO CELL FUNCTION ALTERATIONS

In pathological conditions, changes in flotillin expression levels can markedly alter their localization and function and have been linked to various diseases. For instance, flotillin overexpression has been observed in various cancer cell types, leading to enhanced cell proliferation, migration, invasion, and metastasis (reviewed in [Gauthier‐Rouvière et al., 2020]). This is explained by their involvement in the clustering and trafficking of key proteins (e.g., growth factor receptors, adhesion molecules, metalloproteinases) that modulate signaling pathways implicated in cell cycle progression, cell adhesion, migration, and extracellular matrix remodeling, and by their influence on the actin cytoskeleton that is essential for the invasive behavior. Upregulated flotillin trafficking (UFIT) pathway is the name we proposed for the flotillin‐mediated endosomal pathway that is upregulated in flotillin‐overexpressing cells. Moreover, changes in the expression of flotillins in neurodegenerative disorders, such as Parkinson's disease and Alzheimer's disease, can affect membrane trafficking and receptor recycling, contributing to the cell dysfunction. In Alzheimer's disease, flotillins are involved in amyloid precursor protein (APP) processing and trafficking, and their dysregulation might lead to the accumulation of beta amyloid (βA) plaques (Angelopoulou et al., 2020). This suggests a role for flotillins in the pathogenesis of this disorder.

Metabolic disorders, including obesity and type 2 diabetes, have also been linked to flotillin dysfunction. Flotillins are involved in insulin signaling and glucose uptake, and their altered expression can disrupt these processes, leading to insulin resistance and metabolic dysregulation (Fecchi et al., 2006; Tsutsumi et al., 2024).

Flotillins also play important roles in pathogen infection in several species (e.g., humans, crustaceans, plants). Flotillins expressed in the host cells facilitate the pathogen's entry, survival, and propagation in the host organism (Korhonen et al., 2012; Schmidt et al., 2020; Shi et al., 2016; Wang et al., 2022; Xiong et al., 2019). Moreover, flotillins in pathogens help to assemble virulence‐related protein complexes and enhance their infectious potential. Recently, Koch et al. investigated flotillin role in the multi‐drug‐resistant pathogen *Staphylococcus aureus* and showed that the flotillin‐homolog protein FloA promotes the oligomerization of membrane protein complexes and assists in the assembly of the degradosome, a protein complex which upregulates the expression of virulence genes in *S. aureus*. Targeting flotillin oligomerization with small molecules decreased *S. aureus* virulence potential in vitro and in vivo (Koch et al., 2017). Furthermore, bacterial FloA interaction with PBP2a, a low‐affinity penicillin‐binding protein responsible for penicillin resistance in methicillin‐resistant *S. aureus*, stabilizes PBP2a and favors its correct folding (Ukleja et al., 2024). It would be interesting to determine whether flotillins play a similar function in eukaryotes due to their association with neurodegenerative diseases and other diseases related to protein misfolding and aggregation (e.g., prion pathologies).

The pathological implications of flotillin dysregulation underscore the potential of their therapeutic targeting in these diseases.

## STRUCTURAL PROPERTIES OF FLOTILLINS ALREADY KNOWN AND RECENTLY REVEALED BY CRYO‐ELECTRON MICROSCOPY

**FIGURE 1:**
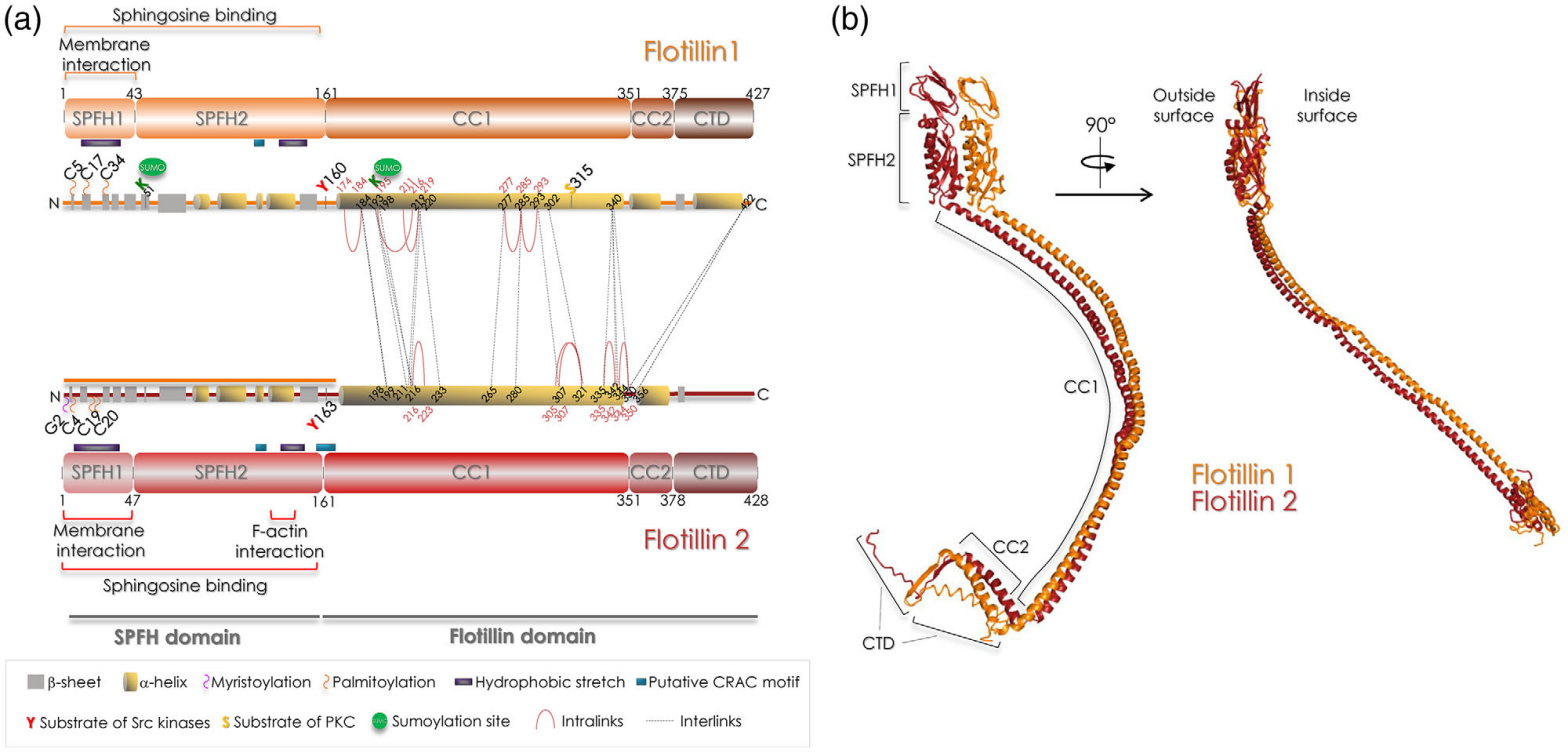
Structure of flotillins. (a) Linear representation of flotillin 1 and 2. The different functional domains are indicated and also some residues (cysteine and glycine residues for palmitoylation and myristoylation, tyrosine residue identified as substrate of Src‐kinase family members, and lysine residues targeted by SUMOylation). Red solid and black dotted lines indicate the DSSO‐induced cross‐links between lysine residues identified in (Singh et al., 2022). These bonds are theoretically generated if the distance between lysine residues is <3.5 nm. The red lines show the intralinks in flotillin 1 and flotillin 2 and the black dotted lines show the interlinks between flotillin 1 and flotillin 2 that have a distance <3.5 nm in the cryo‐EM structure of the 44‐mer flotillin oligomer. (b) Structure of a flotillin1/2 heterodimer extracted from the 44‐mer oligomer visualized by cryo‐EM (Fu & MacKinnon, 2024) (see Figure 2). The outside and inside surfaces indicate the dimer faces exposed toward the outside and the inside of the flotillin cage shown in Figure 2. DSSO, disuccinimidyl sulfoxide; EM, electron microscopy.

Metazoan flotillin 1 and 2, on which we focus in the review, are associated with the cytosolic leaflet of cell membranes and are 427 and 428 amino acids (aa)‐long proteins, respectively. Recent studies (Fu & MacKinnon, 2024; Lu et al., 2024; Singh et al., 2022) established that their polypeptidic chains are organized into three parts (Figure 1). The first part is the SPFH domain (aa 1–160 in both flotillins), further divided into the SPFH1 domain (aa 1–43 in flotillin 1 and aa 1–47 in flotillin 2) and the SPFH2 domain (aa 43–160 in flotillin 1 and aa 48–160 in flotillin 2). The SPFH1 domain is crucial for membrane attachment because the N‐terminus harbors palmitoylated and myristoylated residues (C34 in flotillin 1 and G2, C4, C19, and C20 in flotillin 2) essential for the association with the membrane and localization in lipid rafts (Morrow et al., 2002; Neumann‐Giesen et al., 2004). Non‐palmitoylated flotillin 1 can undergo SUMOylation at K51 and K195 that promotes its nuclear translocation (Jang et al., 2019). Additionally, the SPFH domains contain two hydrophobic stretches (aa 10–35 and aa 134–151 in flotillin 1; aa 7–37 and aa 135–150 in flotillin 2) likely to mediate interactions with lipids (Rivera‐Milla et al., 2006).

The second part is a coiled‐coil domain (CC1) of 190 residues (aa 161–351 in both flotillins) that is significantly longer than in other SPFH family members. The C‐terminus (C) (aa 349–427 in flotillin 1 and aa 352–428 in flotillin 2) includes an alpha‐helix (CC2), followed by a short beta‐strand ending in the C‐terminal domain (CTD) that consists of an alpha‐helix in flotillin 1 and an unstructured region in flotillin 2.

In cells, flotillins form hetero‐oligomers, but their precise organization remained unclear until recent advancements. Stuermer's group (Solis et al., 2007) identified highly stable hetero‐tetramers, made of two flotillin 1 and two flotillin 2 molecules, as potential building blocks of larger oligomers. They showed that the CC2 domain and to a lesser extent, the CC1 domain are necessary for tetramer formation.

In the last two years, three studies have significantly advanced our understanding of flotillin oligomers and their interactions with cell membranes (Fu & MacKinnon, 2024; Lu et al., 2024; Singh et al., 2022). By using chemical cross‐linking between lysine residues and mass spectrometry, we identified 22 interlinks between flotillin 1 and 2 and also 11 intralinks within each protein, all with distances <3.5 nm. Most interlinks between flotillin 1 and 2 are located in the CC1 region (aa 193–365 in flotillin 1 and aa 213–362 in flotillin 2) (Singh et al., 2022). Based on the inter‐ and intra‐links and the detection of flotillin oligomers over 1 MDa, a model of a 38‐mer structure was built that comprises 19 flotillin 1 and 2 dimers (Singh et al., 2022). The 3.7 MDa rat liver major vault protein complex formed by the assembly of the major vault protein was used as a template because it shares several structural properties with flotillins (Lu et al., 2024). This in silico ring‐shaped model was particularly interesting because of its high degree of similarity with the crystal structure obtained by the group of N Gao for the supramolecular complex formed by 12 dimers of HflK/C, two bacterial proteins of the SPFH superfamily (Ma et al., 2022).

**FIGURE 2:**
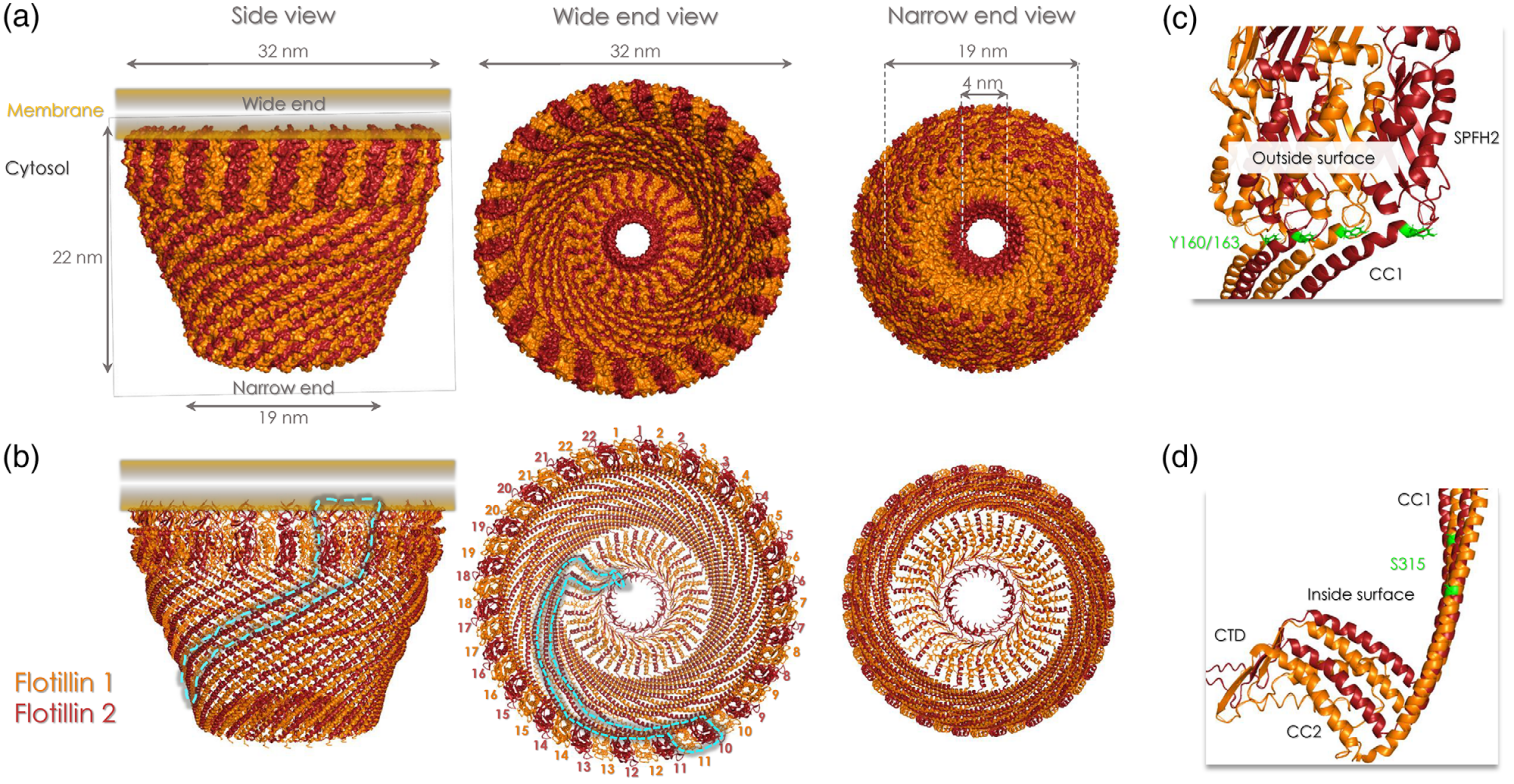
Overall structure of the flotillin oligomer. (a) From left to right: views from the side, the wide end and the narrow end (cytoplasmic view) of the cryo‐EM map obtained by R MacKinnon's group (Fu & MacKinnon, 2024). The dimensions of the complex are indicated (height, diameters of the wide and narrow ends and also of the hole formed by the C‐terminal regions). (b) From left to right: views from the side, the wide end and the narrow and of the corresponding atomic model (Fu & MacKinnon, 2024). In A and B, the cellular membrane is in orange to show how the SPFH1 regions are embedded into the cytosolic leaflet (determined by cryo‐EM). The 22 copies of each flotillin are numbered. The cyan dashed lines delineate one flotillin 1/2 dimer within a whole flotillin complex from both side and base views. Amino acids 423–427 of flotillin 1 and amino acids 403–428 of flotillin 2 are not included in the shown cryo‐EM structure. The figure was generated thanks to Z. Fu and R. MacKinnon who shared the cryo‐EM map file of the flotillin oligomer. (c,d) Zoomed views of the SPFH2/CC1 interface showing that Y160 and Y163 are accessible on the outer surface of the flotillin oligomer (c) and that S315 in the CC1 of flotillin 1 is oriented toward the inside surface of the flotillin oligomer (d).SPFH, stomatin, prohibitin, flotillin, HflK/C; EM, electron microscopy.

This year (2024), using cryo‐electron microscopy (cryo‐EM) approaches, the groups of R MacKinnon (Fu & MacKinnon, 2024) and Q Guo and N Gao (Lu et al., 2024) confirmed the circular cage‐organized flotillin oligomer model we anticipated. Both groups obtained structures, at an overall resolution of 3.5 Å, revealing, for the first time, a complex of 22 copies of flotillin 1 and 2 dimers that co‐assemble to form a truncated cone‐shaped cage (Figure 2a,b). This conical cage has a wide end (diameter of approximately 32 nm), a narrow end (diameter of about 19 nm) and a height of 22 nm. In the cryo‐EM model, the distances between the lysine residues involved in the identified inter‐ and intra‐links are, as expected, < 3.5 nm (Singh et al., 2022). Remarkably, these flotillin structures were observed in their native membrane environment from plasma membrane‐derived vesicles (Fu & MacKinnon, 2024) and in situ by cryo‐electron tomography and cryo‐correlative light and electron microscopy (CLEM) (Lu et al., 2024). The SPFH domains form the large circular rim of this 44‐mer truncated cone‐shaped structure with an external size of 32 nm. Interestingly, as they are oriented nearly perpendicular to the membrane, only the SPFH1 subregion, which contains the palmitoylated/myristoylated residues and the first hydrophobic stretch, is inserted into the cytosolic leaflet. The involvement of the second hydrophobic stretch seems excluded in this type of organization because it is located too far from the membrane. However, as the resolution of the SPFH region was low in the obtained 44‐oligomer structure, it is questionable whether the involvement of the SPFH2 domain in membrane interaction can be truly excluded. It remains to be determined whether it could participate in membrane interactions in another type of flotillin oligomer organization. The CC1 domains are organized into a single layer of 44 parallel‐arranged helices creating a right‐handed helical barrel, named the wall region. These helices are composed of repeated aa patterns characterized by charged residues both inside and outside the α‐helical barrel and allow the formation of hydrogen bonds that glue adjacent subunits together. The C‐terminus (aa 349–427 in flotillin 1 and aa 352–428 in flotillin 2) forms the narrow end of the cone‐shaped cage and its various subdomains are folded to create a hole with a diameter of 4 nm at its center. The electron micrographs obtained by Fu et al. suggest that the narrow‐end region can interact with membranes (Fu & MacKinnon, 2024). Interestingly, a very similar structure, called the outer membrane dome protein complex (OMDP), was previously observed by cryo‐CLEM at the limiting membrane of lamellar bodies and multivesicular endosomes (MVEs) in A549 lung cells (Klein et al., 2021). Although the proteins forming this OMDP were not identified in this study, they might correspond to the 44‐mer flotillin structure described by the MacKinnon's and Gao's groups because the shape and size are identical. This hypothesis is supported also by the fact that flotillins are present in compartments of the late endosome family to which lamellar bodies and MVEs belong.

## THE DISCOVERY OF FLOTILLIN OLIGOMERS SHEDS NEW LIGHT ON THEIR FUNCTIONS

These new findings give a realistic idea of how flotillins assemble and therefore, help to better understand and propose new hypotheses on how they may influence protein complexes and membrane remodeling.

It is interesting to note that several proteins of the SPFH family have now been shown to form circular oligomers. For instance, this is the case of HflK/C (Ma et al., 2022), flotillins (Fu & MacKinnon, 2024; Lu et al., 2024), stomatin (Fu & MacKinnon, 2024; Yokoyama & Matsui, 2023), and also very likely prohibitin (Lange et al., 2024). All these proteins are anchored to the cell membranes. This property may favor the formation of protein oligomers, since once associated with the membrane protein diffusion is limited to two dimensions. Whether the oligomerization of these SPFH proteins requires membrane anchoring remains to be determined, the observation of membrane‐free oligomers has not been reported. Unlike FloA, HfLK/C, prohibitin or stomatins, flotillins have no hydrophobic intramembrane domain, and their membrane binding relies solely on myristoylation and palmitoylation, the latter being reversible and favoring their affinity for liquid ordered domains where protein diffusion is reduced. This suggests that the assembly of flotillin oligomers could be highly regulated by palmitoylation and depalmitoylation and favored in membrane organelles containing sphingolipid and/or cholesterol‐rich liquid ordered domains.

Two tyrosine residues (Y160 on flotillin 1 and Y163 on flotillin 2), which can be phosphorylated by Src‐kinases and are located at the hinge between the SPFH and CC1 domains (Figure 2c), are essential for flotillin‐mediated endocytosis of the GPI‐anchored CD59 glycoprotein and for the EGF‐induced translocation of flotillins from the plasma membrane to late endosomes (Riento et al., 2009). The exact effect of these phosphorylation sites on flotillin oligomers remains to be elucidated. Interestingly, in the recent model (Singh et al., 2022) and the structures revealed by cryo‐EM (Fu & MacKinnon, 2024; Lu et al., 2024), Y160 and Y163 seem quite accessible at the outer surface of flotillin oligomers (Figure 2c). It is not clear whether their phosphorylation can directly influence the interactions between flotillins to modulate oligomer assembly or disassembly. Given the accessibility of these tyrosine residues at the external surface, they might also represent an anchoring site for membrane or cytosolic proteins that could indirectly modulate the formation of the oligomers and consequently, their scaffolding properties and functions.

Moreover, according to the cryo‐EM structure, the PKC‐phosphorylated site on S315 of flotillin 1 (Cremona et al., 2011) faces the internal face of the oligomer (Figure 2d). This site could regulate the flotillin oligomer shape, or dynamic assembly, or the association of flotillin with the sodium‐dependent dopamine transporter (DAT) because phosphorylation of this site is essential for flotillin‐mediated DAT internalization induced by PKC activation (Kobayashi et al., 2019).

Flotillins are involved in membrane remodeling, notably by generating clathrin‐independent endocytic sites (Frick et al., 2007). However, it is unclear how the 44‐mer flotillin cages influence membrane shape. Fu et al. proposed that phosphorylation at Y160 and Y163 affects the SPFH region bending in the flotillin oligomer, thus influencing the curvature of the associated membrane and initiating endocytosis (Fu & MacKinnon, 2024). However, so far, there is no evidence of such mechanism. In both cryo‐EM‐based approaches, the observed flotillin oligomers in which the SPFH domain rings were associated with flat or negatively curved membranes were of a single size (Fu & MacKinnon, 2024; Klein et al., 2021; Lu et al., 2024). It can be hypothesized that 44‐mer flotillin structures may transiently form larger oligomers that can remodel membranes, although biophysical constraints could limit them to 22 flotillin 1/flotillin 2 heterodimers. Alternatively, 44‐mer flotillin structures could act cooperatively in groups to sculpt membranes. Notably, Lu et al. observed 44‐mer flotillin structure clusters that formed microdomains over 200 nm in diameter on various membranes (plasma membrane, endosomes, extracellular vesicles) (Lu et al., 2024), potentially serving as scaffolds for membrane remodeling and endocytic site formation at the plasma membrane. This clustering resembles the assembly of caveolin‐1 disks in caveola formation, although flotillin cages are twice as large than caveolin disks and not flat (Porta et al., 2022). Given their affinity for cholesterol and sphingolipid microdomains, flotillins and caveolins may remodel membranes in similar lipid environments.

The lateral assembly of flotillin cages might involve intermediate partners, including actin filaments. The SPFH domain of flotillin 2 binds to F‐actin via the α4 helix (aa 126–145) (Langhorst et al., 2007) that, according to the obtained cryo‐EM structure, is accessible because it is exposed at the cage's external surface. Additionally, other proteins associated with the actin cytoskeleton, such as vinexin and CAP/ponsin, as well as the cadherin adhesive complex‐associated nectin and afadin proteins can bind to flotillins through their sorbin homology (SoHo) domain (Kimura et al., 2001), potentially contributing to flotillin oligomer clustering.

Flotillin assembly might also involve recently discovered direct flotillin partners such as the ubiquitously expressed membrane palmitoylated protein 1 (MPP1) (Biernatowska et al., 2021, 2022) and EFR3A (Trybus et al., 2023). MPP1 interacts with flotillins 1 and 2 through an amino‐acid motif within its D5 domain. Molecular dynamic simulations predict that this MPP1‐ amino‐acid motif interacts with the CC1 region of both flotillins (amino acids 251–55 in flotillin 1 and 205–210 in flotillin 2) (Biernatowska et al., 2021). The importance of this interaction in flotillin assembly is supported by observations showing that in erythroid cells, flotillins and MPP1 colocalize at the plasma membrane in clusters of a few hundred nanometers, and that downregulation of MPP1 increases the lateral mobility of flotillins at the plasma membrane and decreases the confinement of sphingomyelin (Biernatowska et al., 2022). EFR3A has been identified as a flotillin 2 binding partner, but the sites involved in this interaction remain to be determined. Observations showing that EFR3A expression silencing decreases membrane lipid order and sphingomyelin confinement suggest that this protein may also act as a factor promoting flotillin assembly (Trybus et al., 2023).

To induce membrane remodeling and endocytosis, flotillin oligomers could also promote the local production of bioactive lipids that influence membrane curvature directly or indirectly by recruiting proteins to the membranes. As discussed below, flotillin may act by favoring sphingosine 1‐phosphate, proposed to promote membrane remodeling (Shen et al., 2014). In addition, PI(4,5)P_2_, known for its role in clathrin‐dependent endocytosis (Posor et al., 2015), might be involved in flotillin‐mediated endocytosis, as flotillins have been shown to participate in the recruitment of a type I phosphatidylinositol‐4‐phosphate 5‐kinase at the plasma membrane of T cell's uropod (Mathis et al., 2013).

Flotillin microdomains may have a size limit. Total internal reflection fluorescence microscopy analysis of HeLa cells that overexpress flotillin 1‐GFP and flotillin 2‐GFP revealed microdomains of approximately 0.5 µm in diameter that contained approximately 175 molecules of each flotillin (1:1 stoichiometry) (Frick et al., 2007). This could represent the assembly of eight 44‐mer or larger flotillin oligomers. Moreover, a CRY2‐CIBN‐based optogenetic approach to induce flotillin‐oligomerization indicated that flotillin‐CIBN‐mCherry microdomains reached diameters of 0.5–1 µm at the plasma membrane after 1 min of illumination, without any increase in size upon prolonged illumination (Genest et al., 2022).

On their initial discovery, flotillins were described as proteins that can scaffold membrane microdomains and participate in the formation of protein complexes in membranes. For instance, flotillin microdomains participate in T‐cell activation by regrouping signaling proteins in the vicinity of the T cell receptor (TCR)/CD3 complex (Langhorst et al., 2006). They are also essential for the formation of functional cadherin/p120 catenin complexes, required for the generation of mature adherent junctions (Guillaume et al., 2013; Taulet et al., 2009). In macrophage phagolysosomes, the assembly of two major defense complexes (i.e., vATPase and NADPH oxidase) requires flotillin‐mediated membrane microdomains (Schmidt et al., 2020). As 44‐mer flotillin structures were observed at the surface of intracellular vesicles and notably MVEs (Klein et al., 2021; Lu et al., 2024), they may also play a role in the assembly of these protein complexes that are also critical for endosome functions.

**FIGURE 3:**
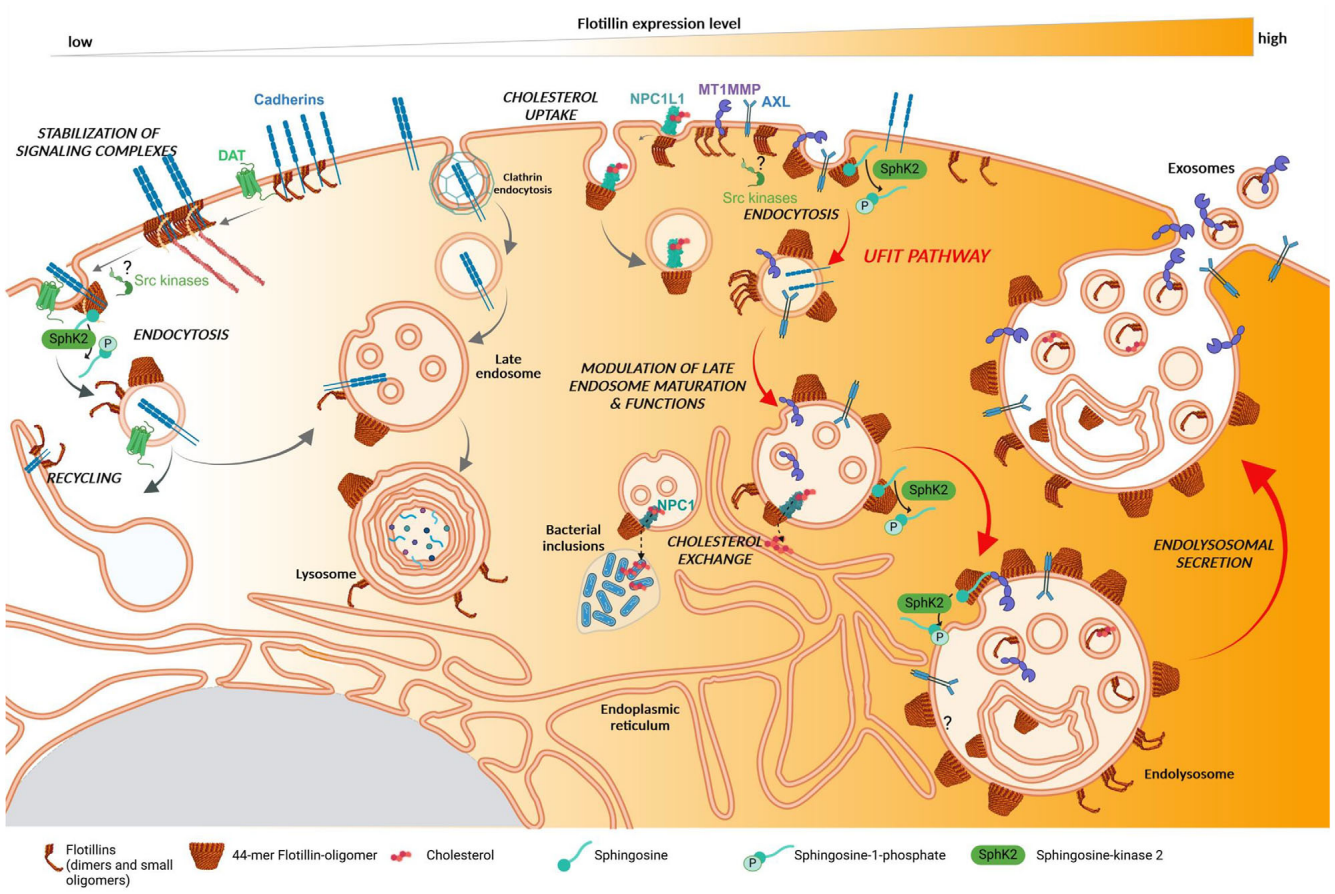
Model depicting flotillin localization and some of their roles in membrane traffic. At physiological expression levels (left part), flotillins participate in scaffolding of membrane domains, such as cadherin‐containing domains at the plasma membrane (Guillaume et al., 2013), and may also participate in membrane remodeling, particularly during clathrin‐independent endocytosis. When flotillin expression levels are increased (right part), as observed in several human diseases, the UFIT (upregulated flotillin trafficking) pathway is switched on. Flotillin role in endocytosis is increased and this favors the endocytosis of several protein cargos toward flotillin‐rich endolysosomes that are not degradative, and participate in the recycling and secretion of protein cargos. These cargos include tyrosine kinase receptors (AXL) (Genest et al., 2022), cholesterol transporters NPC1L1 (Niemann–Pick C1‐like 1) (Ge et al., 2011), and the metalloproteinase MT1‐MMP (membrane type 1 matrix metalloproteinase) (Planchon et al., 2018). The figure also shows the interaction of flotillins with NPC1 (Niemann–Pick C1) located in endolysosomes and their reported role in cholesterol transfer to bacterial (*Anaplasma phagocytophilum)* inclusions (Huang et al., 2021; Xiong et al., 2019). On the top right, the figure shows that flotillins can participate in the secretion of protein cargos and cholesterol via exosomes (Strauss et al., 2010). Flotillin binding to sphingosine promotes the scaffolding of sphingosine‐rich membrane domains that recruit SphK2 both at the plasma membrane and on flotillin‐rich endolysosomes. The figure shows the described 44‐mer flotillin cages and also other possible flotillin intermediates the existence of which needs to be confirmed using powerful techniques, such as cryo‐EM. The 44‐mer flotillin structure could act in scaffolding, membrane remodeling, contact with other membrane organelles, and fusion. EM, electron microscopy.

The structures visualized by cryo‐EM lead us to ask how flotillins organize proteins into membranes. They raise the important question of whether transmembrane receptors in the vicinity of the flotillin cages are localized inside or outside these structures. If these cages are involved in the formation of active complexes, it is more likely that the receptors are present on the outside because inside the flotillin cage, they could not interact with cytosolic proteins. As flotillins mediate endocytosis, another hypothesis could be that flotillin tetramers first pre‐assemble in oligomers that help to form functional signaling complexes. Then, these oligomers might mature into cages that trap receptors to promote their endocytosis and downregulate their signaling activity (Figure 3). Several cargos of the UFIT pathway mentioned in Gauthier‐Rouviere et al. (Gauthier‐Rouvière et al., 2020) could be subjected to such a mechanism, as well as latrophilins, G protein‐coupled receptors that are enriched in the sub‐population of flotillin‐positive endosomes (Singh et al., 2022).

However, it cannot be excluded that for some protein complexes, being concentrated inside the flotillin cages is not beneficial because this would prevent their interaction with other protein partners. This was suggested for the bacterial flotillin homologs HflK/C that also form a corral around the membrane‐anchored AAA+ protease FtsH (Ma et al., 2022). (Ma et al., 2022). It would also be interesting to determine whether flotillin cages trap proteins to facilitate their folding because, as mentioned above this function was recently reported for bacterial FloA (Ukleja et al., 2024).

## FLOTILLINS AND CELLULAR CHOLESTEROL HOMEOSTASIS

Since their discovery as two of the few proteins enriched in isolated lipid rafts (Bickel et al., 1997), flotillins have been considered proteins with high affinity for cholesterol‐ and sphingolipid‐enriched microdomains. Several studies suggest that flotillins can directly bind to cholesterol due to the presence of putative cholesterol‐recognizing amino acid consensus (CRAC) motifs in their SPFH domain (Huang et al., 2021; Rai et al., 2016; Roitbak et al., 2005, 2005; Strauss et al., 2010). Flotillin 1 contains one CRAC motif (aa 117–124, VEEIYKDR) and flotillin 2 has two CRAC motifs (aa 120–127, VEQIYQDR and aa 157–169, VYDKVDYLSSLGK) located outside the two hydrophobic stretches that, according to the cryo‐EM structure, reside outside the surface of the flotillin cage part that interacts with membranes (Fu & MacKinnon, 2024; Lu et al., 2024). Unlike caveolin, which directly interacts with cholesterol (Yang et al., 2014), flotillin binding to cholesterol in vitro remains untested. Several studies suggest that flotillins may not bind to cholesterol. First, their presence in isolated lipid rafts is unaffected by cholesterol depletion, unlike caveolin (Biernatowska et al., 2013; Jang et al., 2013). Additionally, ceramide‐induced‐cholesterol displacement from lipid rafts significantly reduces caveolin presence, with minimal effect on flotillins (Yu et al., 2005). Lastly, flotillins were not among the 250 cholesterol‐interacting proteins identified by a chemoproteomic approach using clickable photoreactive sterol probes (Hulce et al., 2013).

Although these studies strongly suggest that flotillins are unlikely to bind to cholesterol, several works have shown that flotillins and cholesterol reciprocally influence their cellular distribution, with critical consequences for the cellular cholesterol homeostasis. First, in the rat hepatic cell line CRL1601, flotillins are involved in cholesterol uptake mediated by the cholesterol transporter Niemann–Pick C1‐like 1 (NPC1L1) (Ge et al., 2011). Flotillins and NPC1L1 constitutively form a complex independent of the cellular cholesterol levels. The association between flotillins and NPC1L1 is abolished by the NPC1L1 inhibitor ezitimibe, a hypocholesterolemic drug. In CRL1601 cells, flotillins are co‐internalized with NPC1L1 in the presence of cholesterol in the medium, indicating their interdependence in endocytosis. Flotillin internalization is abolished by cholesterol depletion, limiting their localization to the plasma membrane. The cholesterol‐dependent localization of flotillin in endosomes could require NPC1L1 expression. However, in the tested cell lines that are not expected to express NPC1L1 (MDA‐MB‐231 breast cancer cells and MCF10A mammary epithelial cells (S Bodin, unpublished data)), flotillin localization at endosomes was not abolished by cholesterol depletion. In CRL1601 cells, flotillins are critical for NPC1L1‐mediated cholesterol uptake. Intriguingly, although flotillins do not colocalize with clathrin and are considered to mediate clathrin‐independent endocytosis (Frick et al., 2007; Glebov et al., 2006; Lang et al., 1998; Otto & Nichols, 2011), they appear to be required to recruit AP2‐clathrin close to NPC1L1 to mediate clathrin‐dependent cholesterol endocytosis (Ge et al., 2011).

Flotillins may also be involved in cholesterol secretion via exosomes (Strauss et al., 2010). In the mouse Oli‐neu oligodendroglial precursor cell line, the distribution of flotillins is sensitive to the cholesterol level (Strauss et al., 2010). In this cell line, flotillins are very abundant in endosomes, but cholesterol depletion limits their presence at the plasma membrane. This suggests that flotillin internalization is cholesterol‐dependent. Flotillins are abundant in exosomes, which are extracellular vesicles secreted from MVEs upon fusion with the plasma membrane (van Niel et al., 2018). Interestingly, their levels in exosomes from Oli‐neu cells are reduced by cholesterol depletion and increased after loading with exogenous cholesterol (Strauss et al., 2010). This indicates that cholesterol regulates flotillin levels in these extracellular vesicles. This modulation may depend on the ability of flotillins to interact with cholesterol because simultaneous mutation of the residues Y124 and Y163, located in the two putative CRAC motifs of flotillin 2, prevents its presence in exosomes. However, cholesterol influence on flotillin sorting into exosomes might be indirect. Given the lack of evidence of the direct binding of flotillins to cholesterol and considering that Y163 is essential for flotillin 2 internalization from the plasma membrane (Riento et al., 2009), the absence of the Y124/163G‐flotillin 2 mutant in exosomes could be explained by the fact that it never reaches MVEs. Conversely, flotillins promote cholesterol accumulation in exosomes of Oli‐neu cells, suggesting that they participate in the regulation of cholesterol homeostasis in the cells by promoting its exosome‐dependent secretion. It remains to be determined whether this secretory route is common to other cell types and whether it is driven by the direct interaction between flotillins and cholesterol, or by a downstream flotillin‐dependent mechanism. In Niemann‐Pick C1‐type disease that is associated with NPC1 deficiency, cholesterol efflux from late endosomes to the endoplasmic reticulum is severely impaired, leading to abnormal intracellular cholesterol accumulation. In pathological conditions, flotillin‐dependent exosomal cholesterol secretion may be important for limiting cholesterol accumulation in late endosomes (Strauss et al., 2010).

More recent studies demonstrated that flotillins actively participate in deregulating the cellular distribution of free cholesterol and that this favors infections by the Gram‐negative obligatory intracellular bacterium *Anaplasma phagocytophilum* (Huang et al., 2021; Xiong et al., 2019). Unlike most bacteria, *A. phagocytophilum* contains in its membrane a substantial amount of free cholesterol that is essential for its survival and growth. In host cells, *A. phagocytophilum* form inclusions around which low‐density lipoprotein (LDL)‐containing vesicles accumulate. Acid‐lipase converts LDL‐carried esterified cholesterol into free cholesterol that is then transferred to bacterial inclusions by hijacking NPC1 located on the vesicles surrounding these inclusions. *A. phagocytophilum* entry into host cells is flotillin‐independent. Conversely, its intracellular growth requires flotillins (Xiong et al., 2019). Flotillins localize to NPC1‐positive vesicles and as previously shown for NPC1L1 (Ge et al., 2011), they interact with NPC1 through their SPFH domain (Huang et al., 2021). This interaction depends on NPC1 ability to bind to cholesterol and is inhibited by ezitimibe. The flotillins/NPC1 complex allows the transfer of free cholesterol from the surrounding vesicles to the bacteria in the inclusions.

Altogether, these studies point to important links between flotillins and the cholesterol transporters NPC1 and NPC1L1. Given their structures and the measurements of the cone‐shaped flotillin oligomers, it could be hypothesized that NPC1 or NPC1L1 is located within the ring formed by the 44 SPFH domains, as shown for the HflK/C oligomer surrounding four FtsH proteases (Ma et al., 2022). As the transmembrane region of NPC1 and NPC1L1 is ∼4 nm wide (Li et al., 2017; Long et al., 2021), one or more copies of these transporters could fit within the 30 nm diameter of the flotillin ring. Moreover, the luminal region of both transporters and the flotillin oligomers are located on the opposite sides of the membrane, reducing spatial constraints within the flotillin cage. Free cholesterol, after crossing the membrane via NPC1 or NPC1L1, could be transported across the flotillin cage and exit through the 4 nm hole at the narrow end that could eventually come into contact with other membranes (Fu & MacKinnon, 2024), notably the endoplasmic reticulum.

## FLOTILLINS BIND TO SPHINGOSINE AND FAVOR THE GENERATION OF SPHINGOSINE 1‐PHOSPHATE

Flotillin link to cholesterol homeostasis is probably indirect through mechanisms based on their association with NPC1 or NPC1L1, key players in cholesterol metabolism. On the other hand, it has recently emerged that flotillins are closely linked to sphingolipid metabolism. In vitro, flotillin 1 and 2 bind to sphingosine directly via their SPFH domain (Riento et al., 2018), but it is not known whether they can bind to other sphingolipids. Based on the visualization of the structure of the flotillin oligomer in its membrane environment (Fu & MacKinnon, 2024; Lu et al., 2024), it seems likely that the SPFH1 subdomain, integrated into the cytosolic leaflet, is involved in the interaction with sphingosine. In agreement with flotillin binding to sphingosine in vitro, upon incubation with ^3^H‐sphingosine, an increase in the incorporated radioactivity was observed in HeLa cells that ectopically overexpress flotillins. Interestingly, flotillins can affect sphingosine cellular distribution. The ectopic localization of flotillin 2 to mitochondria in cells incubated with ^3^H‐ sphingosine resulted in increased radioactivity in the isolated mitochondrial fraction. Moreover, when control mouse embryonic fibroblasts (MEFs) and flotillin 1^‐/‐^ MEFs were incubated with ^3^H‐sphingosine, the proportion of radioactive lipids in isolated lipid rafts was drastically reduced in flotillin 1^‐/‐^ MEFs (Riento et al., 2018). In contrast, when similar experiments were performed using ^3^H‐cholesterol, no change was observed.

Sphingosine is the central lipid in sphingolipid metabolism, notably serving as the precursor of two main bioactive lipids: ceramides and sphingosine‐1‐phosphate (S1P) that are synthesized by ceramide synthases and sphingosine kinases (SphK), respectively (Green et al., 2021). Comparative lipidomic analyses performed using control MEFs and MEFs in which flotillins were knocked out and also different tissues from control mice and flotillin 1^‐/‐^ / flotillin 2^‐/‐^ mice revealed a decrease in S1P without changes in the other tested lipids, notably sphingosine (Riento et al., 2009). Reciprocally, flotillin overexpression in MCF10A mammary epithelial cells led to an increase in S1P levels compared with parental cells (Genest et al., 2022).

By binding to sphingosine, flotillin oligomers may concentrate sphingosine molecules within the membrane microdomains they scaffold at the plasma membrane and in endosomes, facilitating S1P generation. Notably, SphK2, but not SphK1, is recruited to flotillin‐rich sites at the plasma membrane and in late endosomes (Genest et al., 2022). This suggests that SphK2 plays a crucial role in flotillin‐mediated endocytosis, supporting the emerging role of S1P in membrane trafficking (Kajimoto et al., 2018, 2013; Shen et al., 2014).

Some mechanisms orchestrated by flotillin oligomers might depend on their binding to sphingosine and on the stimulation of S1P production. Future studies should assess whether flotillins modulate the levels of other sphingolipids. S1P is a bioactive sphingolipid that acts in many cellular processes (e.g., signaling, histone acetylation, mitochondrial functions, autophagy and vesicular trafficking) (Cartier & Hla, 2019). Thus, modulating flotillin expression may influence many mechanisms through S1P generation.

## INFLUENCE OF FLOTILLINS ON MEMBRANE TRAFFICKING AND DISEASES

To date, the role of flotillins in cell functions has been studied mainly using loss‐of‐function approaches. Knockdown or knockout of flotillins has demonstrated their role in the establishment of protein complexes at the plasma membrane and in endocytosis. Flotillins are essential in zebrafish during early development (Morris et al., 2017). Conversely, flotillin‐deficient mice are viable and fertile (Bitsikas et al., 2014; Ludwig et al., 2010). This suggests that compensatory mechanisms may exist to overcome flotillin deficiency, preventing a comprehensive view of all flotillin‐regulated cellular processes when using only flotillin‐deficient biological models. Interestingly, flotillins are overexpressed in some human pathologies, such as cancer, Alzheimer's disease, and Parkinson's disease. Studying the impact of flotillin upregulation, as observed in these pathological contexts, shed new light on the cellular mechanisms in which flotillins play a role.

When upregulated, flotillins have a gain‐of‐function effect and modify the vesicular trafficking toward an endocytic‐endolysosomal secretory pathway, which we called the UFIT pathway (Figure 3). This pathway was clearly demonstrated in the tumor context (Gauthier‐Rouvière et al., 2020; Genest et al., 2022; Planchon et al., 2018) and seems to occur also in neurodegenerative disorders, such as Parkinson's and Alzheimer's diseases (Angelopoulou et al., 2020). As we have previously reviewed the implication of the UFIT pathway in the deregulation of protein cargos in tumor cells (Gauthier‐Rouvière et al., 2020), we decided to focus here on other pathological contexts. Indeed, among the cargos of the flotillin‐mediated endocytic route, several proteins are deregulated in Alzheimer's disease, particularly APP, and in Parkinson's disease, particularly DAT, glutamate transporter, and α‐synuclein (Cremona et al., 2011; Kim et al., 2016; Kobayashi et al., 2019; Schneider et al., 2008).

In Parkinson's disease, two key pathogenic processes are associated with flotillins. The first is perturbation of neurotransmitter receptor localization, particularly DAT decrease from the cell surface. The second is α‐synuclein accumulation in endolysosomes. Flotillin 1 is highly expressed in the brainstem catecholaminergic neurons and is strongly upregulated in Parkinson's disease (Jacobowitz & Kallarakal, 2004). Flotillins and notably flotillin 1 palmitoylation are essential for DAT presence in lipid rafts and for its endocytosis (Cremona et al., 2011). PKC phosphorylates flotillin 1 on S315 and triggers DAT internalization. However, it is unknown whether this phosphorylation affects flotillin oligomerization or its association with DAT. Interestingly, flotillin 1‐induced endocytic trafficking of DAT might be involved in the buildup of Rab7‐positive α‐synuclein aggregates and, consequently, in the formation of Lewy bodies that are involved in the loss of catecholaminergic neurons (Kobayashi et al., 2019). The UFIT pathway implication in Parkinson's disease is also suggested by the distribution of internalized α‐synuclein preformed fibrils in endolysosomes and exosomes (Bayati et al., 2022). According to another mechanism that relies on flotillins but not on their upregulation, Parkinson's disease‐associated DJ‐1/PARK7 deficiency is associated with flotillin 1 decrease and altered glutamate endocytosis that could exploit the role of flotillins as scaffolds of lipid raft domains (Kim et al., 2016).

Accumulating evidence suggests a role for flotillins in Alzheimer's disease (reviewed in (Angelopoulou et al., 2020). This disorder is characterized by the accumulation of Aβ, a 40 or 42 aa peptide, in extracellular senile plaques, and of intracellular neurofibrillary tangles that mainly consist of tau proteins. Several studies reported increased flotillin levels in brain samples from patients with Alzheimer's disease (Girardot et al., 2003; Kokubo et al., 2000). These studies demonstrated a direct interaction of flotillin 1 with the intracellular domain of amyloidogenic APP (Chen et al., 2006) and with BACE1/gamma‐secretase, which is involved in Aβ peptide production (John et al., 2014). Again, the UFIT pathway could participate in APP clustering, endocytosis, processing, and release through exosomes (Bitsikas et al., 2014; John et al., 2014; Rajendran et al., 2007; Schneider et al., 2008). Aβ accumulates in endolysosomes, the compartment where flotillins accumulate when upregulated (Willén et al., 2017). Interestingly, Alzheimer's disease is associated with SphK2, a key player of the UFIT pathway, that unlike SphK1, is upregulated in brain samples from patients with Alzheimer's disease, and as a result leads to S1P overproduction in neurons (Takasugi et al., 2011). The authors reported that S1P directly activates BACE1, the rate‐limiting step in Aβ peptide production.

A role for flotillins in the regulation of cargo‐sorting events within endosomes and the recycling process has been described in T‐cell activation (Redpath et al., 2019), in tumor cells (Solis et al., 2013), and also in infections. In zebrafish, flotillin's role in retrograde transport and cargo recycling was proposed for sorting cholera toxin‐GM1 complexes from endosomes via the trans‐Golgi network to the endoplasmic reticulum (Saslowsky et al., 2010). The depletion of both flotillins makes the fish resistant to cholera toxin intoxication. Also, in cultured cells, flotillin depletion does not affect the endocytic uptake of ricin and Shiga toxin but impairs their retrograde transport toward the trans Golgi network and the endoplasmic reticulum, causing their accumulation and increasing their toxicity (Pust et al., 2010).

In tumor cells, flotillin overexpression affects signaling complex assembly and activation, and the UFIT pathway is responsible for the endocytosis of proteins, such as the metalloproteinase MT1‐MMP (Planchon et al., 2018) and the tyrosine kinase receptor AXL (Genest et al., 2022), toward flotillin‐positive late endosomes. Then, these cargos could be exocytosed and recycled at the cell surface to participate in tumorigenesis (Figure 3). Hijacking the trafficking of these cargoes by the UFIT pathway alters their activity and participates in invasive tumorigenesis. Using the powerful emerging cryo‐CLEM technology to detect these UFIT pathway cargos at the plasma membrane and in endolysosomes could give interesting information on the organization of flotillin oligomers during the clustering and trafficking of these cargos.

## CONCLUSIONS AND PERSPECTIVES

Flotillins are pivotal players in cellular processes, particularly in sphingolipid metabolism and membrane trafficking, where their interactions with key lipids and proteins place them at the heart of crucial signaling pathways and cellular homeostasis. Their upregulation in various diseases, including cancer and neurodegenerative disorders, underscores their importance in disease progression and highlights their potential as therapeutic targets.

The latest findings on the oligomerization of flotillins into a 44‐mer ring structure provide insight on their mechanisms of action but also raise critical questions on how these complexes segregate proteins, promote endocytosis, and remodel membranes. Whether this 44‐mer structure represents the basic structural unit that could evolve into larger structures remains to be determined. Indeed, the dynamic nature of lipid rafts (size ranging from 10 to 300 nm) suggests that 44‐mer flotillin structures, of a size of 30 nm, may cluster to form microdomains. Depending on the size of these microdomains, the protein partners could be different, engaging flotillins in different cellular processes. Moreover, it is not known whether intermediate flotillin clusters, smaller than the 44‐mer cage, are present in cells. Therefore, it would be of great interest to explore using cryo‐EM the formation of flotillin cages and their possible intermediate stages in cells with altered flotillin levels and after direct manipulation of flotillin clustering using established photoactivation protocols (Genest et al., 2022).

The observation of flotillin oligomers on different membranes (plasma membranes, endosomes of the endolysosomal family, and exosomes) agrees with their described function in these compartments. How these complexes participate in flotillin‐mediated endocytosis is an open question, as well as how they participate in inter‐membrane trafficking, because flotillin‐mediated cone‐shaped structures can bridge vesicles (Fu & MacKinnon, 2024).

One consequence of the UFIT pathway is the accumulation of flotillin‐positive intracellular vesicles in the late endosomal compartment, particularly in endolysosomes. Currently, the role of flotillins in these intracellular vesicles is poorly described. Emerging evidence indicates that S1P is a positive regulator of contact sites between late endosomes and the endoplasmic reticulum involved in NPC1‐independent cholesterol egress from endolysosomes. This suggests that flotillin oligomers may play a role in lipid exchange between organelles (Newton et al., 2020; Palladino et al., 2022). More studies on this relationship could reveal new insights into lipid transport in cells.

As scaffolding proteins in lipid rafts, flotillins facilitate membrane‐initiated signaling by clustering proteins. Investigating whether flotillins in eukaryotes, like their bacterial counterparts, stabilize unfolded proteins could enhance our understanding of their role in neurodegenerative diseases associated with protein misfolding and aggregation.

Future research should focus on elucidating the molecular mechanisms by which flotillins regulate sphingolipid metabolism and membrane dynamics. Understanding the structure and function of flotillin oligomers will be crucial for uncovering their role in lipid microdomain organization and cargo trafficking. As we continue to unravel the complexities of flotillin function, integrating advanced imaging techniques and high‐throughput lipidomic analyses will enhance our understanding of their contributions to cell physiology and pathology. Ultimately, a comprehensive knowledge of flotillin biology may offer innovative strategies for the diagnosis and treatment of diseases characterized by disrupted lipid metabolism and altered cellular trafficking.

## AKNOWLEDGMENTS

We thank Ziao Fiu and Roderick MacKinnon (The Rockefeller University, New York, USA) for sharing with us the cryo‐EM density map of the flotillin complex. This work was financially supported by the Institut National du Cancer (INCa) and Ligue contre la cancer. C.G.R. was financially supported by the Institut national de la Santé et de la Recherche Médicale (INSERM). The authors acknowledge that other studies on flotillins may exist besides those cited in this review. The Figure 3 and graphical abstract were created in Biorender (https://BioRender.com/g26p547 for Figure 3; https://BioRender.com/y03w478 for the graphical abstract).

## CONFLICT OF INTEREST STATEMENT

The authors declare no conflicts of interest.

## Data Availability

Data sharing is not applicable to this article as no new data were created or analyzed in this study.

## References

[boc202400134-bib-0001] Affentranger, S. , Martinelli, S. , Hahn, J. , Rossy, J. & Niggli, V. (2011) Dynamic reorganization of flotillins in chemokine‐stimulated human T‐lymphocytes. BMC Cell Biology, 12, 28. 10.1186/1471-2121-12-28 21696602 PMC3131241

[boc202400134-bib-0002] Angelopoulou, E. , Paudel, Y.N. , Shaikh, Mohd.F. & Piperi, C. (2020) Flotillin: a promising biomarker for Alzheimer's disease. Journal of Personalized Medicine, 10(2), 20. 10.3390/jpm10020020 32225073 PMC7354424

[boc202400134-bib-0003] Babuke, T. , Ruonala, M. , Meister, M. , Amaddii, M. , Genzler, C. , Esposito, A. & Tikkanen, R. (2009) Hetero‐oligomerization of reggie‐1/flotillin‐2 and reggie‐2/flotillin‐1 is required for their endocytosis. Cellular Signalling, 21, 1287–1297. 10.1016/j.cellsig.2009.03.012 19318123

[boc202400134-bib-0004] Banning, A. , Babuke, T. , Kurrle, N. , Meister, M. , Ruonala, M.O. & Tikkanen, R. (2018) Flotillins regulate focal adhesions by interacting with α‐actinin and by influencing the activation of focal adhesion kinase. Cells, 7(4), 28. 10.3390/cells7040028 29642469 PMC5946105

[boc202400134-bib-0005] Bayati, A. , Banks, E. , Han, C. , Luo, W. , Reintsch, W.E. , Zorca, C.E. , Shlaifer, I. , Del Cid Pellitero, E. , Vanderperre, B. , McBride, H.M. , Fon, E.A. , Durcan, T.M. & McPherson, P.S. (2022) Rapid macropinocytic transfer of α‐synuclein to lysosomes. Cell Reports, 40(3), 111102. 10.1016/j.celrep.2022.111102 35858558

[boc202400134-bib-0006] Bickel, P.E. , Scherer, P.E. , Schnitzer, J.E. , Oh, P. , Lisanti, M.P. & Lodish, H.F. (1997) Flotillin and epidermal surface antigen define a new family of caveolae‐associated integral membrane proteins. Journal of Biological Chemistry, 272, 13793–13802. 9153235 10.1074/jbc.272.21.13793

[boc202400134-bib-0007] Biernatowska, A. , Olszewska, P. , Grzymajło, K. , Drabik, D. , Kraszewski, S. , Sikorski, A.F. & Czogalla, A. (2021) Molecular characterization of direct interactions between MPP1 and flotillins. Scientific Reports, 11(1), 14751. 10.1038/s41598-021-93982-3 34285255 PMC8292550

[boc202400134-bib-0008] Biernatowska, A. , Podkalicka, J. , Majkowski, M. , Hryniewicz‐Jankowska, A. , Augoff, K. , Kozak, K. , Korzeniewski, J. & Sikorski, A.F. (2013) The role of MPP1/p55 and its palmitoylation in resting state raft organization in HEL cells. Biochimica et Biophysica Acta (BBA)—Molecular Cell Research, 1833(8), 1876–1884. 10.1016/j.bbamcr.2013.03.009 23507198

[boc202400134-bib-0009] Biernatowska, A. , Wójtowicz, K. , Trombik, T. , Sikorski, A.F. & Czogalla, A. (2022) MPP1 determines the mobility of flotillins and controls the confinement of raft‐associated molecules. Cells, 11(3), 311. 10.3390/cells11030311 35159121 PMC8834348

[boc202400134-bib-0010] Bitsikas, V. , Riento, K. , Howe, J.D. , Barry, N.P. & Nichols, B.J. (2014) The role of flotillins in regulating Aβ production, investigated using flotillin 1‐/‐, flotillin 2‐/‐ double knockout mice. PLoS One, 9(1), e85217. 10.1371/journal.pone.0085217 24465508 PMC3897416

[boc202400134-bib-0011] Bodin, S. , Planchon, D. , Rios Morris, E. , Comunale, F. & Gauthier‐Rouviere, C. (2014) Flotillins in intercellular adhesion—from cellular physiology to human diseases. Journal of Cell Science, 127, 5139–5147. 10.1242/jcs.159764 25413346

[boc202400134-bib-0012] Cartier, A. & Hla, T. (2019) Sphingosine 1‐phosphate: lipid signaling in pathology and therapy. Science, 366(6463), eaar5551. 10.1126/science.aar5551 31624181 PMC7661103

[boc202400134-bib-0013] Chen, T.‐Y. , Liu, P.‐H. , Ruan, C.‐T. , Chiu, L. & Kung, F.‐L. (2006) The intracellular domain of amyloid precursor protein interacts with flotillin‐1, a lipid raft protein. Biochemical and Biophysical Research Communications, 342(1), 266–272. 10.1016/j.bbrc.2006.01.156 16480949

[boc202400134-bib-0014] Cremona, M.L. , Matthies, H.J.G. , Pau, K. , Bowton, E. , Speed, N. , Lute, B.J. , Anderson, M. , Sen, N. , Robertson, S.D. , Vaughan, R.A. , Rothman, J.E. , Galli, A. , Javitch, J.A. & Yamamoto, A. (2011) Flotillin‐1 is essential for PKC‐triggered endocytosis and membrane microdomain localization of DAT. Nature Neuroscience, 14(4), 469–477. 10.1038/nn.2781 21399631 PMC3066276

[boc202400134-bib-0015] Dermine, J.F. , Duclos, S. , Garin, J. , St‐Louis, F. , Rea, S. , Parton, R.G. & Desjardins, M. (2001) Flotillin‐1‐enriched lipid raft domains accumulate on maturing phagosomes. The Journal of Biological Chemistry, 276(21), 18507–18512. 10.1074/jbc.M101113200 11279173

[boc202400134-bib-0016] Fecchi, K. , Volonte, D. , Hezel, M.P. , Schmeck, K. & Galbiati, F. (2006) Spatial and temporal regulation of GLUT4 translocation by flotillin‐1 and caveolin‐3 in skeletal muscle cells. FASEB Journal, 20, 705–707. 10.1096/05-4661fje 16455755 PMC4288748

[boc202400134-bib-0017] Frick, M. , Bright, N.A. , Riento, K. , Bray, A. , Merrified, C. & Nichols, B.J. (2007) Coassembly of flotillins induces formation of membrane microdomains, membrane curvature, and vesicle budding. Current Biology, 17, 1151–1156. 10.1016/j.cub.2007.05.078 17600709

[boc202400134-bib-0018] Fu, Z. & MacKinnon, R. (2024) Structure of the flotillin complex in a native membrane environment. Proceedings of the National Academy of Sciences, 121(29), e2409334121. 10.1073/pnas.2409334121 PMC11260169 38985763

[boc202400134-bib-0019] Gauthier‐Rouvière, C. , Bodin, S. , Comunale, F. & Planchon, D. (2020) Flotillin membrane domains in cancer. Cancer and Metastasis Reviews, 39(2), 361–374. 10.1007/s10555-020-09873-y 32297092 PMC7311376

[boc202400134-bib-0020] Ge, L. , Qi, W. , Wang, L.‐J. , Miao, H.‐H. , Qu, Y.‐X. , Li, B.‐L. & Song, B.‐L. (2011) Flotillins play an essential role in Niemann‐Pick C1‐like 1‐mediated cholesterol uptake. Proceedings of the National Academy of Sciences, 108(2), 551–556. 10.1073/pnas.1014434108 PMC3021008 21187433

[boc202400134-bib-0021] Genest, M. , Comunale, F. , Planchon, D. , Govindin, P. , Noly, D. , Vacher, S. , Bièche, I. , Robert, B. , Malhotra, H. , Schoenit, A. , Tashireva, L.A. , Casas, J. , Gauthier‐Rouvière, C. & Bodin, S. (2022) Upregulated flotillins and sphingosine kinase 2 derail AXL vesicular traffic to promote epithelial‐mesenchymal transition. Journal of Cell Science, 135(7), jcs259178. 10.1242/jcs.259178 35394045

[boc202400134-bib-0022] Girardot, N. , Allinquant, B. , Langui, D. , Laquerrière, A. , Dubois, B. , Hauw, J.‐J. & Duyckaerts, C. (2003) Accumulation of flotillin‐1 in tangle‐bearing neurones of Alzheimer's disease. Neuropathology and Applied Neurobiology, 29(5), 451–461. 10.1046/j.1365-2990.2003.00479.x 14507337

[boc202400134-bib-0023] Glebov, O.O. , Bright, N.A. & Nichols, B.J. (2006) Flotillin‐1 defines a clathrin‐independent endocytic pathway in mammalian cells. Nature Cell Biology, 8, 46–54. 10.1038/ncb1342 16341206

[boc202400134-bib-0024] Green, C.D. , Maceyka, M. , Cowart, L.A. & Spiegel, S. (2021) Sphingolipids in metabolic disease: the good, the bad, and the unknown. Cell Metabolism, 33(7), 1293–1306. 10.1016/j.cmet.2021.06.006 34233172 PMC8269961

[boc202400134-bib-0025] Guillaume, E. , Comunale, F. , Do Khoa, N. , Planchon, D. , Bodin, S. & Gauthier‐Rouviere, C. (2013) Flotillin microdomains stabilize cadherins at cell‐cell junctions. Journal of Cell Science, 126, 5293–5304. 10.1242/jcs.133975 24046456

[boc202400134-bib-0026] Huang, W. , Xiong, Q. , Lin, M. & Rikihisa, Y. (2021) Anaplasma phagocytophilum hijacks flotillin and NPC1 complex to acquire intracellular cholesterol for proliferation, which can be inhibited with ezetimibe. mBio, 12(5), e0229921. 10.1128/mBio.02299-21 34544283 PMC8546544

[boc202400134-bib-0027] Hulce, J.J. , Cognetta, A.B. , Niphakis, M.J. , Tully, S.E. & Cravatt, B.F. (2013) Proteome‐wide mapping of cholesterol‐interacting proteins in mammalian cells. Nature Methods, 10(3), 259–264. 10.1038/nmeth.2368 23396283 PMC3601559

[boc202400134-bib-0028] Jacobowitz, D.M. & Kallarakal, A.T. (2004) Flotillin‐1 in the substantia nigra of the Parkinson brain and a predominant localization in catecholaminergic nerves in the rat brain. Neurotoxicity Research, 6(4), 245–257. 10.1007/BF03033435 15545008

[boc202400134-bib-0029] Jang, D. , Kwon, H. , Choi, M. , Lee, J. & Pak, Y. (2019) Sumoylation of flotillin‐1 promotes EMT in metastatic prostate cancer by suppressing Snail degradation. Oncogene, 38(17), 3248–3260. 10.1038/s41388-018-0641-1 30631151 PMC6756018

[boc202400134-bib-0030] Jang, D. , Kwon, H. , Jeong, K. , Lee, J. & Pak, Y. (2013) Essential role of flotillin‐1 palmitoylation in the intracellular localization and signaling function of IGF‐1 receptor. Journal of Cell Science, 128(11), 2179–2190. 10.1242/jcs.169409 25908865

[boc202400134-bib-0031] John, B.A. , Meister, M. , Banning, A. & Tikkanen, R. (2014) Flotillins bind to the dileucine sorting motif of β‐site amyloid precursor protein‐cleaving enzyme 1 and influence its endosomal sorting. The FEBS Journal, 281(8), 2074–2087. 10.1111/febs.12763 24612608

[boc202400134-bib-0032] Kajimoto, T. , Mohamed, N.N.I. , Badawy, S.M.M. , Matovelo, S.A. , Hirase, M. , Nakamura, S. , Yoshida, D. , Okada, T. , Ijuin, T. & Nakamura, S. (2018) Involvement of Gβγ subunits of G _i_ protein coupled with S1P receptor on multivesicular endosomes in F‐actin formation and cargo sorting into exosomes. Journal of Biological Chemistry, 293(1), 245–253. 10.1074/jbc.M117.808733 29133526 PMC5766922

[boc202400134-bib-0033] Kajimoto, T. , Okada, T. , Miya, S. , Zhang, L. & Nakamura, S. (2013) Ongoing activation of sphingosine 1‐phosphate receptors mediates maturation of exosomal multivesicular endosomes. Nature Communications, 4(1), 2712. 10.1038/ncomms3712 24231649

[boc202400134-bib-0034] Kim, J.‐M. , Cha, S.‐H. , Choi, Y.R. , Jou, I. , Joe, E.‐H. & Park, S.M. (2016) DJ‐1 deficiency impairs glutamate uptake into astrocytes via the regulation of flotillin‐1 and caveolin‐1 expression. Scientific Reports, 6(1), 28823. 10.1038/srep28823 27346864 PMC4922019

[boc202400134-bib-0035] Kimura, A. , Baumann, C.A. , Chiang, S.‐H. & Saltiel, A.R. (2001) The sorbin homology domain: a motif for the targeting of proteins to lipid rafts. Proceedings of the National Academy of Sciences, 98(16), 9098–9103. 10.1073/pnas.151252898 PMC55379 11481476

[boc202400134-bib-0036] Klein, S. , Wimmer, B.H. , Winter, S.L. , Kolovou, A. , Laketa, V. & Chlanda, P. (2021) Post‐correlation on‐lamella cryo‐CLEM reveals the membrane architecture of lamellar bodies. Communications Biology, 4(1), 137. 10.1038/s42003-020-01567-z 33514845 PMC7846596

[boc202400134-bib-0037] Kobayashi, J. , Hasegawa, T. , Sugeno, N. , Yoshida, S. , Akiyama, T. , Fujimori, K. , Hatakeyama, H. , Miki, Y. , Tomiyama, A. , Kawata, Y. , Fukuda, M. , Kawahata, I. , Yamakuni, T. , Ezura, M. , Kikuchi, A. , Baba, T. , Takeda, A. , Kanzaki, M. , Wakabayashi, K. , … Aoki, M. (2019) Extracellular α‐synuclein enters dopaminergic cells by modulating flotillin‐1–assisted dopamine transporter endocytosis. The FASEB Journal, 33(9), 10240–10256. 10.1096/fj.201802051R 31211923

[boc202400134-bib-0038] Koch, G. , Wermser, C. , Acosta, I.C. , Kricks, L. , Stengel, S.T. , Yepes, A. & Lopez, D. (2017) Attenuating Staphylococcus aureus virulence by targeting flotillin protein scaffold activity. Cell Chemical Biology, 24(7), 845–857.e6. 10.1016/j.chembiol.2017.05.027 28669526 PMC5536197

[boc202400134-bib-0039] Kokubo, H. , Lemere, C.A. & Yamaguchi, H. (2000) Localization of flotillins in human brain and their accumulation with the progression of Alzheimer's disease pathology. Neuroscience Letters, 290(2), 93–96. 10.1016/S0304-3940(00)01334-3 10936685

[boc202400134-bib-0040] Korhonen, J.T. , Puolakkainen, M. , Häivälä, R. , Penttilä, T. , Haveri, A. , Markkula, E. & Lahesmaa, R. (2012) Flotillin‐1 (reggie‐2) contributes to Chlamydia pneumoniae growth and is associated with bacterial inclusion. Infection and Immunity, 80(3), 1072–1078. 10.1128/IAI.05528-11 22215737 PMC3294677

[boc202400134-bib-0041] Lang, D.M. , Lommel, S. , Jung, M. , Ankerhold, R. , Petrausch, B. , Laessing, U. , Wiechers, M.F. , Plattner, H. & Stuermer, C.A. (1998) Identification of reggie‐1 and reggie‐2 as plasmamembrane‐associated proteins which cocluster with activated GPI‐anchored cell adhesion molecules in non‐caveolar micropatches in neurons. Journal of Neurobiology, 37(4), 502–523. 9858255 10.1002/(sici)1097-4695(199812)37:4<502::aid-neu2>3.0.co;2-s

[boc202400134-bib-0042] Lange, F. , Ratz, M. , Dohrke, J.‐N. , Wenzel, D. , Ilgen, P. , Riedel, D. & Jakobs, S. (2024) In‐situ architecture of the human prohibitin complex . 10.1101/2024.02.14.579514 PMC11991916 40119201

[boc202400134-bib-0043] Langhorst, M.F. , Reuter, A. , Jaeger, F.A. , Wippich, F.M. , Luxenhofer, G. , Plattner, H. & Stuermer, C.A.O. (2008) Trafficking of the microdomain scaffolding protein reggie‐1/flotillin‐2. European Journal of Cell Biology, 87(4), 211–226. 10.1016/j.ejcb.2007.12.001 18237819

[boc202400134-bib-0044] Langhorst, M.F. , Reuter, A. , Luxenhofer, G. , Boneberg, E.M. , Legler, D.F. , Plattner, H. & Stuermer, C.A. (2006) Preformed reggie/flotillin caps: stable priming platforms for macrodomain assembly in T cells. FASEB Journal, 20, 711–713. 10.1096/05-4760fje 16452278

[boc202400134-bib-0045] Langhorst, M.F. , Reuter, A. & Stuermer, C.A.O. (2005) Scaffolding microdomains and beyond: the function of reggie/flotillin proteins. Cellular and Molecular Life Sciences, 62(19‑20), 2228–2240. 10.1007/s00018-005-5166-4 16091845 PMC11139094

[boc202400134-bib-0046] Langhorst, M.F. , Solis, G.P. , Hannbeck, S. , Plattner, H. & Stuermer, C.A. (2007) Linking membrane microdomains to the cytoskeleton: regulation of the lateral mobility of reggie‐1/flotillin‐2 by interaction with actin. FEBS Letters, 581, 4697–4703. 10.1016/S0014-5793(07)00961-1 17854803

[boc202400134-bib-0047] Li, X. , Lu, F. , Trinh, M.N. , Schmiege, P. , Seemann, J. , Wang, J. & Blobel, G. (2017) 3.3 Å structure of Niemann–Pick C1 protein reveals insights into the function of the C‐terminal luminal domain in cholesterol transport. Proceedings of the National Academy of Sciences, 114(34), 9116–9121. 10.1073/pnas.1711716114 PMC5576846 28784760

[boc202400134-bib-0048] Long, T. , Liu, Y. , Qin, Y. , DeBose‐Boyd, R.A. & Li, X. (2021) Structures of dimeric human NPC1L1 provide insight into mechanisms for cholesterol absorption. Science Advances, 7(34), eabh3997. 10.1126/sciadv.abh3997 34407950 PMC8373123

[boc202400134-bib-0049] Lu, M.‐A. , Qian, Y. , Ma, L. , Guo, Q. & Gao, N. (2024) Molecular mechanism of the flotillin complex in membrane microdomain organization . 10.1101/2024.05.25.595881

[boc202400134-bib-0050] Ludwig, A. , Otto, G.P. , Riento, K. , Hams, E. , Fallon, P.G. & Nichols, B.J. (2010) Flotillin microdomains interact with the cortical cytoskeleton to control uropod formation and neutrophil recruitment. Journal of Cell Biology, 191, 771–781. 10.1083/jcb.201005140 21059848 PMC2983060

[boc202400134-bib-0051] Ma, C. , Wang, C. , Luo, D. , Yan, L. , Yang, W. , Li, N. & Gao, N. (2022) Structural insights into the membrane microdomain organization by SPFH family proteins. Cell Research, 32(2), 176–189. 10.1038/s41422-021-00598-3 34975153 PMC8807802

[boc202400134-bib-0052] Mathis, L. , Wernimont, S. , Affentranger, S. , Huttenlocher, A. & Niggli, V. (2013) Determinants of phosphatidylinositol‐4‐phosphate 5‐kinase type Iγ90 uropod location in T‐lymphocytes and its role in uropod formation. PeerJ, 1, e131. 10.7717/peerj.131 24010013 PMC3757496

[boc202400134-bib-0053] Morris, E.A.R. , Bodin, S. , Delaval, B. , Comunale, F. , Georget, V. , Costa, M.L. , Lutfalla, G. & Gauthier‐Rouvière, C. (2017) Flotillins control zebrafish epiboly through their role in cadherin‐mediated cell‐cell adhesion: flotillins are required for epiboly in zebrafish. Biology of the Cell, 109(5), 210–221. 10.1111/boc.201700001 28225561

[boc202400134-bib-0054] Morrow, I.C. , Rea, S. , Martin, S. , Prior, I.A. , Prohaska, R. , Hancock, J.F. , James, D.E. & Parton, R.G. (2002) Flotillin‐1/reggie‐2 traffics to surface raft domains via a novel golgi‐independent pathway. Identification of a novel membrane targeting domain and a role for palmitoylation. Journal of Biological Chemistry, 277, 48834–48841. 10.1074/jbc.M209082200 12370178

[boc202400134-bib-0055] Munderloh, C. , Solis, G.P. , Bodrikov, V. , Jaeger, F.A. , Wiechers, M. , Malaga‐Trillo, E. & Stuermer, C.A. (2009) Reggies/flotillins regulate retinal axon regeneration in the zebrafish optic nerve and differentiation of hippocampal and N2a neurons. Journal of Neuroscience, 29, 6607–6615. 10.1523/JNEUROSCI.0870-09.2009 19458231 PMC6665911

[boc202400134-bib-0056] Neumann‐Giesen, C. , Falkenbach, B. , Beicht, P. , Claasen, S. , Luers, G. , Stuermer, C.A. , Herzog, V. & Tikkanen, R. (2004) Membrane and raft association of reggie‐1/flotillin‐2: role of myristoylation, palmitoylation and oligomerization and induction of filopodia by overexpression. Biochemical Journal, 378, 509–518. 10.1042/BJ20031100 14599293 PMC1223955

[boc202400134-bib-0057] Neumann‐Giesen, C. , Fernow, I. , Amaddii, M. & Tikkanen, R. (2007) Role of EGF‐induced tyrosine phosphorylation of reggie‐1/flotillin‐2 in cell spreading and signaling to the actin cytoskeleton. Journal of Cell Science, 120, 395–406. 10.1242/jcs.03336 17213334

[boc202400134-bib-0058] Newton, J. , Palladino, E.N.D. , Weigel, C. , Maceyka, M. , Gräler, M.H. , Senkal, C.E. , Enriz, R.D. , Marvanova, P. , Jampilek, J. , Lima, S. , Milstien, S. & Spiegel, S. (2020) Targeting defective sphingosine kinase 1 in Niemann–Pick type C disease with an activator mitigates cholesterol accumulation. Journal of Biological Chemistry, 295(27), 9121–9133. 10.1074/jbc.RA120.012659 32385114 PMC7335787

[boc202400134-bib-0059] Otto, G.P. & Nichols, B.J. (2011) The roles of flotillin microdomains—Endocytosis and beyond. Journal of Cell Science, 124(23), 3933–3940. 10.1242/jcs.092015 22194304

[boc202400134-bib-0060] Palladino, E.N.D. , Bernas, T. , Green, C.D. , Weigel, C. , Singh, S.K. , Senkal, C.E. , Martello, A. , Kennelly, J.P. , Bieberich, E. , Tontonoz, P. , Ford, D.A. , Milstien, S. , Eden, E.R. & Spiegel, S. (2022) Sphingosine kinases regulate ER contacts with late endocytic organelles and cholesterol trafficking. Proceedings of the National Academy of Sciences, 119(39), e2204396119. 10.1073/pnas.2204396119 PMC9522378 36122218

[boc202400134-bib-0061] Planchon, D. , Rios Morris, E. , Genest, M. , Comunale, F. , Vacher, S. , Bièche, I. , Denisov, E.V. , Tashireva, L.A. , Perelmuter, V.M. , Linder, S. , Chavrier, P. , Bodin, S. & Gauthier‐Rouvière, C. (2018) MT1‐MMP targeting to endolysosomes is mediated by upregulation of flotillins. Journal of Cell Science, 131(17), jcs218925. 10.1242/jcs.218925 30111578

[boc202400134-bib-0062] Porta, J.C. , Han, B. , Gulsevin, A. , Chung, J.M. , Peskova, Y. , Connolly, S. , Mchaourab, H.S. , Meiler, J. , Karakas, E. , Kenworthy, A.K. & Ohi, M.D. (2022) Molecular architecture of the human caveolin‐1 complex. Science Advances, 8(19), eabn7232. 10.1126/sciadv.abn7232 35544577 PMC9094659

[boc202400134-bib-0063] Posor, Y. , Eichhorn‐Grünig, M. & Haucke, V. (2015) Phosphoinositides in endocytosis. Biochimica et Biophysica Acta (BBA)—Molecular and Cell Biology of Lipids, 1851(6), 794–804. 10.1016/j.bbalip.2014.09.014 25264171

[boc202400134-bib-0064] Pust, S. , Dyve, A.B. , Torgersen, M.L. , Van Deurs, B. & Sandvig, K. (2010) Interplay between Toxin Transport and Flotillin Localization. PLoS One, 5(1), e8844. 10.1371/journal.pone.0008844 20107503 PMC2809741

[boc202400134-bib-0065] Rai, A. , Pathak, D. , Thakur, S. , Singh, S. , Dubey, A.K. & Mallik, R. (2016) Dynein clusters into lipid microdomains on phagosomes to drive rapid transport toward lysosomes. Cell, 164(4), 722–734. 10.1016/j.cell.2015.12.054 26853472 PMC4752818

[boc202400134-bib-0066] Rajendran, L. , Knobloch, M. , Geiger, K.D. , Dienel, S. , Nitsch, R. , Simons, K. & Konietzko, U. (2007) Increased Aβ production leads to intracellular accumulation of Aβ in flotillin‐1‐positive endosomes. Neurodegenerative Diseases, 4(2‑3), 164–170. 10.1159/000101841 17596711

[boc202400134-bib-0067] Redpath, G.M.I. , Ecker, M. , Kapoor‐Kaushik, N. , Vartoukian, H. , Carnell, M. , Kempe, D. , Biro, M. , Ariotti, N. & Rossy, J. (2019) Flotillins promote T cell receptor sorting through a fast Rab5–Rab11 endocytic recycling axis. Nature Communications, 10(1), 4392. 10.1038/s41467-019-12352-w PMC6763463 31558725

[boc202400134-bib-0068] Riento, K. , Frick, M. , Schafer, I. & Nichols, B.J. (2009) Endocytosis of flotillin‐1 and flotillin‐2 is regulated by Fyn kinase. Journal of Cell Science, 122(7), 912–918. 10.1242/jcs.039024 19258392 PMC2871078

[boc202400134-bib-0069] Riento, K. , Zhang, Q. , Clark, J. , Begum, F. , Stephens, E. , Wakelam, M.J. & Nichols, B.J. (2018) Flotillin proteins recruit sphingosine to membranes and maintain cellular sphingosine‐1‐phosphate levels. PLoS One, 13(5), e0197401. 10.1371/journal.pone.0197401 29787576 PMC5963794

[boc202400134-bib-0070] Rivera‐Milla, E. , Stuermer, C.A. & Malaga‐Trillo, E. (2006) Ancient origin of reggie (flotillin), reggie‐like, and other lipid‐raft proteins: convergent evolution of the SPFH domain. Cellular and Molecular Life Sciences, 63, 343–357. 10.1007/s00018-005-5434-3 16389450 PMC11135987

[boc202400134-bib-0071] Roitbak, T. , Surviladze, Z. , Tikkanen, R. & Wandinger‐Ness, A. (2005) A polycystin multiprotein complex constitutes a cholesterol‐containing signalling microdomain in human kidney epithelia. Biochemical Journal, 392, 29–38. 10.1042/BJ20050645 16038619 PMC1317661

[boc202400134-bib-0072] Rossy, J. , Schlicht, D. , Engelhardt, B. & Niggli, V. (2009) Flotillins interact with PSGL‐1 in neutrophils and, upon stimulation, rapidly organize into membrane domains subsequently accumulating in the uropod. PLoS One, 4, e5403. 10.1371/journal.pone.0005403 19404397 PMC2671458

[boc202400134-bib-0073] Saslowsky, D.E. , Cho, J.A. , Chinnapen, H. , Massol, R.H. , Chinnapen, D.J.‐F. , Wagner, J.S. , De Luca, H.E. , Kam, W. , Paw, B.H. & Lencer, W.I. (2010) Intoxication of zebrafish and mammalian cells by cholera toxin depends on the flotillin/reggie proteins but not Derlin‐1 or ‐2. Journal of Clinical Investigation, 120(12), 4399–4409. 10.1172/JCI42958 21041954 PMC2994338

[boc202400134-bib-0074] Schmidt, F. , Thywißen, A. , Goldmann, M. , Cunha, C. , Cseresnyés, Z. , Schmidt, H. , Rafiq, M. , Galiani, S. , Gräler, M.H. , Chamilos, G. , Lacerda, J.F. , Campos, A. , Eggeling, C. , Figge, M.T. , Heinekamp, T. , Filler, S.G. , Carvalho, A. & Brakhage, A.A. (2020) Flotillin‐dependent membrane microdomains are required for functional phagolysosomes against fungal infections. Cell Reports, 32(7), 108017. 10.1016/j.celrep.2020.108017 32814035 PMC10054021

[boc202400134-bib-0075] Schneider, A. , Rajendran, L. , Honsho, M. , Gralle, M. , Donnert, G. , Wouters, F. , Hell, S.W. & Simons, M. (2008) Flotillin‐dependent clustering of the amyloid precursor protein regulates its endocytosis and amyloidogenic processing in neurons. Journal of Neuroscience, 28(11), 2874–2882. 10.1523/JNEUROSCI.5345-07.2008 18337418 PMC6670660

[boc202400134-bib-0076] Schulte, T. , Paschke, K.A. , Laessing, U. , Lottspeich, F. & Stuermer, C.A. (1997) Reggie‐1 and reggie‐2, two cell surface proteins expressed by retinal ganglion cells during axon regeneration. Development, 124, 577–587. 10.1242/dev.124.2.577 9053333

[boc202400134-bib-0077] Shen, H. , Giordano, F. , Wu, Y. , Chan, J. , Zhu, C. , Milosevic, I. , Wu, X. , Yao, K. , Chen, B. , Baumgart, T. , Sieburth, D. & De Camilli, P. (2014) Coupling between endocytosis and sphingosine kinase 1 recruitment. Nature Cell Biology, 16(7), 652–662. 10.1038/ncb2987 24929359 PMC4230894

[boc202400134-bib-0078] Shi, H. , Guo, G. , Liu, R. , Wang, C. , Xu, X. & Ruan, L. (2016) Membrane associated protein flotillin‐2 in Litopenaeus vannamei plays a role in WSSV infection. Fish & Shellfish Immunology, 54, 247–253. 10.1016/j.fsi.2016.04.010 27079424

[boc202400134-bib-0080] Singh, J. , Elhabashy, H. , Muthukottiappan, P. , Stepath, M. , Eisenacher, M. , Kohlbacher, O. , Gieselmann, V. & Winter, D. (2022) Cross‐linking of the endolysosomal system reveals potential flotillin structures and cargo. Nature Communications, 13(1), 6212. 10.1038/s41467-022-33951-0 PMC9584938 36266287

[boc202400134-bib-0081] Solis, G.P. , Hoegg, M. , Munderloh, C. , Schrock, Y. , Malaga‐Trillo, E. , Rivera‐Milla, E. & Stuermer, C.A. (2007) Reggie/flotillin proteins are organized into stable tetramers in membrane microdomains. Biochemical Journal, 403, 313–322. 10.1042/BJ20061686 17206938 PMC1874235

[boc202400134-bib-0082] Solis, G.P. , Hulsbusch, N. , Radon, Y. , Katanaev, V.L. , Plattner, H. & Stuermer, C.A. (2013) Reggies/flotillins interact with Rab11a and SNX4 at the tubulovesicular recycling compartment and function in transferrin receptor and E‐cadherin trafficking. Molecular Biology of the Cell, 24, 2689–2702. 10.1091/mbc.E12-12-0854 23825023 PMC3756921

[boc202400134-bib-0083] Strauss, K. , Goebel, C. , Runz, H. , Mobius, W. , Weiss, S. , Feussner, I. , Simons, M. & Schneider, A. (2010) Exosome secretion ameliorates lysosomal storage of cholesterol in Niemann–Pick type C disease. Journal of Biological Chemistry, 285, 26279–26288. 10.1074/jbc.M110.134775 20554533 PMC2924046

[boc202400134-bib-0084] Stuermer, C.A. (2010) The reggie/flotillin connection to growth. Trends in Cell Biology, 20, 6–13. 10.1016/j.tcb.2009.10.003 19896850

[boc202400134-bib-0085] Sugawara, Y. , Nishii, H. , Takahashi, T. , Yamauchi, J. , Mizuno, N. , Tago, K. & Itoh, H. (2007) The lipid raft proteins flotillins/reggies interact with Gαq and are involved in Gq‐mediated p38 mitogen‐activated protein kinase activation through tyrosine kinase. Cellular Signalling, 19(6), 1301–1308. 10.1016/j.cellsig.2007.01.012 17307333

[boc202400134-bib-0086] Takasugi, N. , Sasaki, T. , Suzuki, K. , Osawa, S. , Isshiki, H. , Hori, Y. , Shimada, N. , Higo, T. , Yokoshima, S. , Fukuyama, T. , Lee, V.M.‐Y. , Trojanowski, J.Q. , Tomita, T. & Iwatsubo, T. (2011) BACE1 Activity Is Modulated by Cell‐Associated Sphingosine‐1‐Phosphate. The Journal of Neuroscience, 31(18), 6850–6857. 10.1523/JNEUROSCI.6467-10.2011 21543615 PMC4534000

[boc202400134-bib-0087] Taulet, N. , Comunale, F. , Favard, C. , Charrasse, S. , Bodin, S. & Gauthier‐Rouviere, C. (2009) N‐cadherin/p120 catenin association at cell‐cell contacts occurs in cholesterol‐rich membrane domains and is required for RhoA activation and myogenesis. Journal of Biological Chemistry, 284, 23137–23145. 10.1074/M109.017665 19546217 PMC2755719

[boc202400134-bib-0088] Tomasovic, A. , Traub, S. & Tikkanen, R. (2012) Molecular networks in FGF signaling: flotillin‐1 and cbl‐associated protein compete for the binding to fibroblast growth factor receptor substrate 2. PLoS One, 7(1), e29739. 10.1371/journal.pone.0029739 22235335 PMC3250484

[boc202400134-bib-0089] Tomiyama, A. , Uekita, T. , Kamata, R. , Sasaki, K. , Takita, J. , Ohira, M. , Nakagawara, A. , Kitanaka, C. , Mori, K. , Yamaguchi, H. & Sakai, R. (2014) Flotillin‐1 regulates oncogenic signaling in neuroblastoma cells by regulating ALK membrane association. Cancer Research, 74(14), 3790–3801. 10.1158/0008-5472.CAN-14-0241 24830726

[boc202400134-bib-0090] Trybus, M. , Hryniewicz‐Jankowska, A. , Wójtowicz, K. , Trombik, T. , Czogalla, A. & Sikorski, A.F. (2023) EFR3A: a new raft domain organizing protein? Cellular & Molecular Biology Letters, 28(1), 86. 10.1186/s11658-023-00497-y 37880612 PMC10601247

[boc202400134-bib-0091] Tsutsumi, R. , Ueberheide, B. , Liang, F.‐X. , Neel, B.G. , Sakai, R. & Saito, Y. (2024) Endocytic vesicles act as vehicles for glucose uptake in response to growth factor stimulation. Nature Communications, 15(1), 2843. 10.1038/s41467-024-46971-9 PMC10987504 38565573

[boc202400134-bib-0092] Ukleja, M. , Kricks, L. , Torrens, G. , Peschiera, I. , Rodrigues‐Lopes, I. , Krupka, M. , García‐Fernández, J. , Melero, R. , Del Campo, R. , Eulalio, A. , Mateus, A. , López‐Bravo, M. , Rico, A.I. , Cava, F. & Lopez, D. (2024) Flotillin‐mediated stabilization of unfolded proteins in bacterial membrane microdomains. Nature Communications, 15(1), 5583. 10.1038/s41467-024-49951-1 PMC11222466 38961085

[boc202400134-bib-0093] van Niel, G. , D'Angelo, G. & Raposo, G. (2018) Shedding light on the cell biology of extracellular vesicles. Nature Reviews Molecular Cell Biology, 19(4), 213–228. 10.1038/nrm.2017.125 29339798

[boc202400134-bib-0094] Volonté, D. , Galbiati, F. , Li, S. , Nishiyama, K. , Okamoto, T. & Lisanti, M.P. (1999) Flotillins/cavatellins are differentially expressed in cells and tissues and form a hetero‐oligomeric complex with caveolins in vivo. Journal of Biological Chemistry, 274(18), 12702–12709. 10.1074/jbc.274.18.12702 10212252

[boc202400134-bib-0095] Wang, W. , Qiao, L. , Lu, H. , Chen, X. , Wang, X. , Yu, J. , Zhu, J. , Xiao, Y. , Ma, Y. , Wu, Y. , Zhao, W. & Cui, F. (2022) Flotillin 2 facilitates the infection of a plant virus in the gut of insect vector. Journal of Virology, 96(7), e0214021. 10.1128/jvi.02140-21 35254088 PMC9006895

[boc202400134-bib-0096] Willén, K. , Edgar, J.R. , Hasegawa, T. , Tanaka, N. , Futter, C.E. & Gouras, G.K. (2017) Aβ accumulation causes MVB enlargement and is modelled by dominant negative VPS4A. Molecular Neurodegeneration, 12(1), 61. 10.1186/s13024-017-0203-y 28835279 PMC5569475

[boc202400134-bib-0097] Xiong, Q. , Lin, M. , Huang, W. & Rikihisa, Y. (2019) Infection by *Anaplasma phagocytophilum* requires recruitment of low‐density lipoprotein cholesterol by flotillins. mBio, 10(2), e02783‐18. 10.1128/mBio.02783-18 30914515 PMC6437059

[boc202400134-bib-0098] Yang, G. , Xu, H. , Li, Z. & Li, F. (2014) Interactions of caveolin‐1 scaffolding and intramembrane regions containing a CRAC motif with cholesterol in lipid bilayers. Biochimica et Biophysica Acta (BBA)—Biomembranes, 1838(10), 2588–2599. 10.1016/j.bbamem.2014.06.018 24998359

[boc202400134-bib-0099] Yokoyama, H. & Matsui, I. (2023) Higher‐order structure formation using refined monomer structures of lipid raft markers, Stomatin, Prohibitin, Flotillin, and hflk/C‐related proteins. FEBS Open Bio, 13(5), 926–937. 10.1002/2211-5463.13593 PMC10153343 36932695

[boc202400134-bib-0100] Yu, C. , Alterman, M. & Dobrowsky, R.T. (2005) Ceramide displaces cholesterol from lipid rafts and decreases the association of the cholesterol binding protein caveolin‐1. Journal of Lipid Research, 46, 1678–1691. 10.1194/jlr.M500060-JLR200 15863835

